# Growth of Romaine Lettuce in Eggshell Powder Mixed Alginate Hydrogel in an Aeroponic System for Water Conservation and Vitamin C Biofortification

**DOI:** 10.3390/gels10050322

**Published:** 2024-05-09

**Authors:** Fariha Afnan, Md Nayeem Hasan Kashem, Rutwik Joshi, Catherine Simpson, Wei Li

**Affiliations:** 1 Department of Chemical Engineering, Texas Tech University, Lubbock, TX 79409, USA; fafnan@ttu.edu (F.A.); md-nayeem-hasan.kashem@ttu.edu (M.N.H.K.); rutwjosh@ttu.edu (R.J.); 2 Department of Plant and Soil Science, Texas Tech University, Lubbock, TX 79409, USA

**Keywords:** vitamin C, biofortification, water conservation, hydrogels, aeroponics, ascorbic acid

## Abstract

Vitamin C is crucial for physical well-being, and its deficiency can lead to severe health consequences. Biofortification has been used to address this deficiency by enhancing vitamin C in plants. Additionally, soilless agriculture has been used to conserve and optimize water use in comparison to conventional agriculture. While hydrogels have been shown to improve water conservation and are used for biofortification in crops, their application has only been explored in soil-based and hydroponic farming. The aeroponics system is a plant-growing method that has shown potential for increasing yields and biomass while conserving water and nutrients. In this paper, we have developed an aeroponic-compatible medium to grow romaine lettuce (*Lactuca sativa* L.) with eggshell powder (ESP) mixed with calcium-alginate hydrogel as a substrate and nutrient source aiming to conserve water and incorporate vitamin C through biofortification. Herein, lower water spray time and higher intervals, with varied gel types and ESP concentrations, resulted in healthy lettuce growth. Plants treated with 0.5% ascorbic acid-absorbed ESP-mixed alginate hydrogel for biofortification showed higher levels of vitamin C compared to the traditional method. This study suggests using an alginate hydrogel–ESP-based substrate in aeroponics to reduce water usage and enhance plant biofortification of vitamin C.

## 1. Introduction

Minerals, vitamins, and nutrients are important aspects of human health. Among the vitamins essential for human health, vitamin C is a significant component of the human diet. Vitamin C is a helpful antioxidant that reduces inflammation and cardiovascular diseases, promotes iron (Fe^2+^) absorption in the gut, and boosts energy [1,2,3,4,5]. Vitamin C is a form of ascorbic acid, and its oxidized form is L-dehydroascorbic acid (DHA). The total vitamin C is calculated as a sum of ascorbic acid and DHA [6]. Vitamin C deficiency can lead to scurvy, which can cause different health conditions such as anemia, infections, bleeding gums, depression, muscle weakness, and fatigue [7,8]. Unfortunately, there is a considerable lack of vitamin C intake in a significant amount of the population, up to 23%. This situation is not only limited to underdeveloped and developing countries but also to developed countries as well.

Humans cannot synthesize vitamin C directly, so the available and easy way to consume vitamin C is through plants, animals, and supplements [9,10]. Vitamin C obtained in plant sources possesses greater bioavailability than other sources [10]. However, the vitamin C concentration varies in plant sources, so a regulated and defined concentration in the plant sources is necessary to obtain the targeted vitamin C concentration. Biofortification is a sustainable method of incorporating micronutrients into food crops, which has been considered helpful in delivering micronutrients to rural populations in developing countries [11]. Biofortification of vitamin C in plants would help reach the daily intake goal as the concentration can be regulated. 

For the biofortification of vitamin C in plants, some of the common methods of implementation include agronomics and bio-engineering [12]. Agronomic biofortification is an inexpensive and widely used technique for the biofortification of minerals by soil and foliar fertilization and soil inoculation. Nonetheless, limited research has been conducted on increasing the vitamin C content in plants through agronomic biofortification. This is due to the fact that the concentration of ascorbic acid in plants varies depending on the time of harvesting and storage, which makes it challenging to achieve a specific target [13,14]. This led to the incorporation of vitamin C into plants through biofortification by bio-engineering [15]. Nevertheless, this process is costly and time-consuming, which could be a concern when producing crops on a larger scale for staple crops but could be more economical for higher-value horticultural crops. So, to address these issues, an achievable and sustainable method is needed to biofortify the ascorbic acid in the plants to mitigate the daily intake goal of vitamin C.

With a view to addressing the agronomic biofortification of vitamin C in plants, soil-based and soilless agriculture have been used as alternatives. Soil-based agriculture requires a vast area of land, inefficient water irrigation, and soil degradation, which leads to soilless growing techniques with higher plant growth efficiency and low water consumption. Soilless methods such as hydroponics and aeroponics systems have gained attention due to their lower water consumption rate than soil-based agriculture and accessibility in urban farming. In particular, hydroponics is a soilless growing technique where plants are grown in nutrient-rich water under controlled conditions with 10% less water consumption than the conventional method [16,17]. However, the plant roots are suspended in nutrient water for their growth, which may lead to the spread of diseases from one plant to another. On the contrary, the aeroponic method is advantageous over hydroponic systems because plants are sprayed with nutrient-rich water through atomizers after specific time intervals [18]. Moreover, the water usage in this technique for plant growth is reduced by almost 98% compared to the conventional soil-based methods. While biofortification of different nutrients has been performed in the aeroponic systems in plants, few studies are available on the biofortification of vitamin C in plants with hydroponics, and to the best of our knowledge, no significant studies were found for aeroponic systems. 

Furthermore, hydrogels are a three-dimensional network system that consists of polymeric chains and water [19]. The water percentage in hydrogels is very high, consisting of more than 90% of their original weight. Due to this high water absorption capacity, both natural and synthetic-based hydrogels have been previously used as a plant culture substrate in agriculture [20,21,22,23,24,25]. For example, macroalgal-derived alginate hydrogels were used for soil amendments with increased water retention capacity [26]. Another example is the development of alginate/polyacrylamide hydrogel, which showed a greater swelling ratio and increased the potential to be applied for plant growth [27]. These studies showed that hydrogel application has improved water retention, enhancing crop growth and nutrient transfer to the plants. 

Hydrogels have also been used as a medium for the biofortification of nutrients in soil-based and soilless agriculture [28,29]. A study by Dutta et al. proposed cellulose-derived hydrogels as biofortification materials for nutrients carried in soil [30]. Another study showed selenium–agarose hybrid hydrogel as a recyclable natural substrate for plant selenium enrichment in a soilless medium [31]. These further pave the way to explore the potential use of hydrogels in aeroponics, which can increase water efficiency and nutrient delivery to plants, minimize water stress experienced in the plant roots, and create scope for the biofortification of minerals and nutrients into the plants through hydrogels. Nonetheless, studies regarding biofortification through hydrogel in aeroponic media were not found.

The objective of this paper is to develop a system that utilizes an aeroponic medium and a support made of alginate hydrogel mixed with eggshell powder to increase vitamin C levels in romaine lettuce while also improving water efficiency and maintaining plant growth. Romaine lettuce is used for being widely used as a leafy green and has a variety of nutrients available that are beneficial for human health [32,33]. Eggshell powder, widely used in agriculture as a fertilizer and nutrient source for plants, has been used with the goal of providing nutrients and support to the plants over time, thus increasing the plant yield [34,35]. Ca-alginate, being a biodegradable hydrogel, has been previously used to increase plant growth, which has improved the water intake of plants [26,36]. Herein, lower water consumption was obtained in an aeroponic system with the ESP-mixed alginate hydrogel acting as a substrate for the root. The growth conditions in the aeroponic system were carefully chosen based on the development criteria of romaine lettuce. The nutrient-rich water spray time was chosen to be 10 s at two-hour intervals. Notably, this spray time is significantly lower, and the interval is significantly higher than that of a conventional aeroponic system, which could further enhance water conservation efforts [37,38,39]. Plant growth was observed for a period of time, and an optimum gel type and ESP concentration were chosen for further studies. Microscopic and SEM image analysis were performed to understand the distribution of roots in the ESP mixed gels. The impact of spray time interval was also studied for plant growth. The vitamin C concentration in lettuce is generally 9 to 24 mg/100 g FW [13,40]. Here, the vitamin C concentration in the plants with the ascorbic acid treatment showed significantly higher values compared to the conventional soil-based method. 

## 2. Results and Discussion

**Figure 1:**
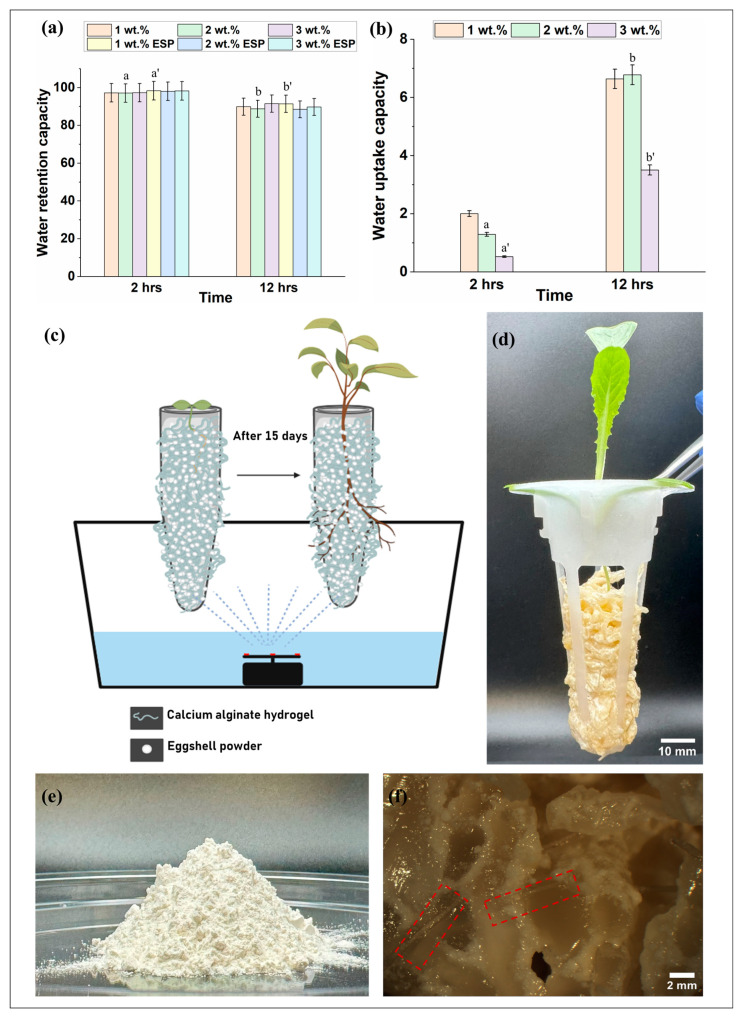
(**a**) Water retention capacity of alginate hydrogels in different conditions. (**b**) Water uptake capacity of alginate hydrogel. (**c**) Schematic of lettuce growing process. (**d**) Romaine lettuce after 15 days in alginate hydrogel. (**e**) Eggshell powder (ESP). (**f**) High-resolution image of root distributed through ESP-mixed-alginate hydrogel taken from MTV-3 camera adapter. Samples marked by different letters are significantly different from one another, while those marked by the same letter are not significantly different (*p* < 0.05; *n* = 3, Students *t*-test was performed).

For optimal plant growth, it is crucial that they have access to moisture in their growth environment. To assess the water retention capacity (WRC) of the hydrogels being utilized to support plant growth, we observed the WRC of crosslinked alginate hydrogels with varying degrees of crosslinking concentration. After 2 h of monitoring (Figure 1a), it could be seen from the graph that the hydrogel samples with and without the ESP have WRC close to 98% and 97%, respectively. This indicates that the crosslinking concentration and the addition of ESP did not have any significant differences in WRC, and the addition of the ESP would not cause the gel to dry by absorbing moisture from it over time.

For the 12 h water retention experiments depicted in Figure 1a, the WRC values dropped from 97~98% to 89~92% for all hydrogel samples due to evaporation after 12 h (*p* < 0.05). Here, the graph also suggests that crosslinking density and the addition of ESP do not significantly affect WRC values. This would further indicate that even after 12 h of evaporation, hydrogel samples, irrespective of ESP addition, did not cause any difference in their WRC. Therefore, the ESP addition would not be responsible for drying the gels at any time intervals, as it was seen in previous studies that the addition of ESP has been beneficial for water absorption properties in polymer composites [41]. So, the addition of ESP would be beneficial for water retention in the Ca-alginate hydrogels as well. Also, the water retention capacity of the bare alginate hydrogels after the given time interval is consistent with the literature [42,43].

The water uptake capacity (WUC) was also determined (see Figure 1b) to understand the ability of hydrogels to absorb water after a certain time interval. The WUC for the hydrogels crosslinked with 1, 2, and 3 wt.% CaCl_2_ after 2 h and 12 h can be seen in Figure 1b. The water uptake capacity (WUC) values obtained after 2 h revealed a maximum value of 2%. It is noteworthy that the gels had already retained moisture by this point, which could potentially explain the low WUC values observed for the samples (See Figure 1a). However, the WUC was lower in the 3 wt.% crosslinked gels compared to the other crosslinked densities after 2 h. Since the crosslinking density is higher in the 3 wt.% crosslinked gels, this could be responsible for the lower water uptake capacity [44]. Moreover, higher WUC values were observed among the gels in 1 and 2 wt.% after 2 and 12 h (*p* < 0.05) and gels between 2 and 12 h for 3 wt.% (*p* = 0.05). This higher WUC can be further validated by the comparatively lower WRC values in the gels after 12 h (Figure 1a). Herein, 1 and 2 wt.% crosslinked Ca-alginate hydrogels also demonstrated better WUC than the 3 wt.% sample. This suggests that crosslinking density influences WUC values. The hydrogels should possess the required mechanical strength to support the weight of the plants and withstand the pressure exerted by the growing roots. According to the literature, an increase in the concentration of CaCl_2_ solution has been found to be positively correlated with an improvement in the mechanical strength of gels [45]. So, the 3 wt.% would have better mechanical strength than 1 and 2 wt.% CaCl_2_ crosslinked gels. Similarly, 2 wt.% crosslinking density would possess better mechanical properties than 1 wt.% crosslinked gels. However, the water retention and water uptake capacity were lower in the 3 wt.% CaCl_2_ crosslinked gels. Based on these, 2 wt.% CaCl_2_ crosslinked alginate hydrogel was chosen for our subsequent studies. 

Afterward, to investigate the growth of romaine lettuce on the selected CaCl_2_ crosslinked hydrogel, different concentrations of eggshell powder (ESP) (Figure 1e) were mixed with Ca-alginate hydrogels, and the germinated seedlings were planted in them. Daily monitoring of the plant’s growth, based on the measurement of leaf area and shoot length, was carried out until day 10. On day 15, the plant exhibited a significant increase in size, with visibly grown green leaves, as depicted in Figure 1d. As the plants grew, their roots distributed themselves into the alginate hydrogels and received nutrients from the nutrient water sprayed on them, as shown in Figure 1f. The red dotted sections are the roots, and the transparent gel-like texture is the Ca-alginate hydrogels after 15 days. The white powder surrounding the gels is eggshell powder. The roots get the moisture needed for the plants from the gel. After 15 days, the plants were carefully taken out of the alginate hydrogels, and the roots and plants were cleaned with deionized water until most of the alginate hydrogels came away from the plant roots. The roots and shoots were carefully separated with a disinfected scalpel, and further measurements were taken. 

**Figure 2:**
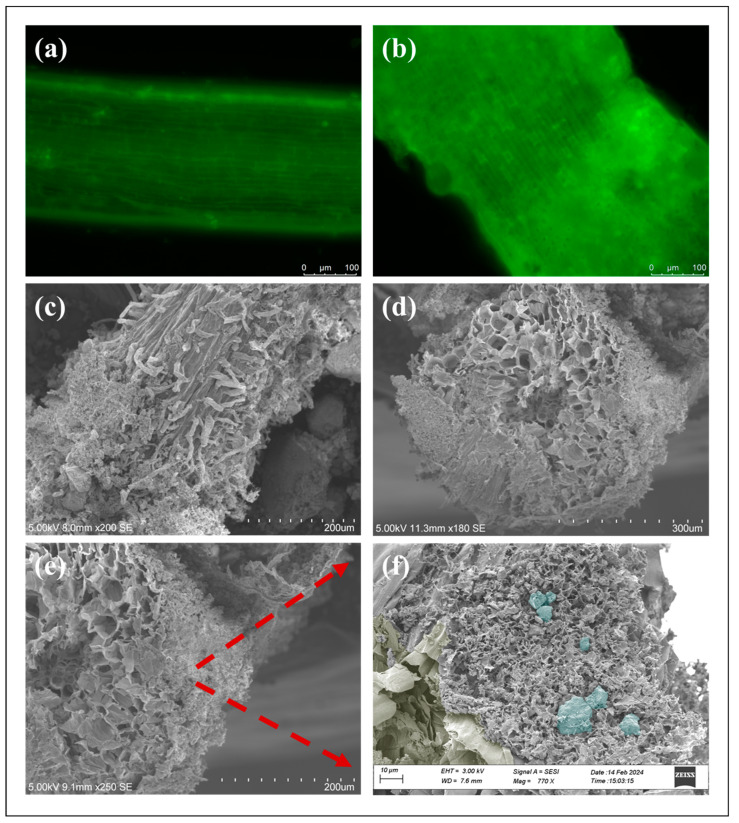
Images of plant root and root with eggshell powder (ESP) mixed alginate hydrogel. (**a**) Microscopic image of the bare plant root; (**b**) microscopic image of plant root with ESP mixed Ca-alginate;(**c**) SEM image of the root surface with alginate-eggshell powder; (**d**–**f**) SEM images of the root cross-section with alginate-eggshell powder.

Microscopic and SEM image analyses were performed to visualize the hydrogel distribution around the plant roots after 15 days. Figure 2a,b shows the microscopic images of the lettuce roots. From Figure 2a, the root epidermis and the vascular cylinder can also be identified. From Figure 2b, the vascular cylinder is not as prominent as seen in Figure 2a due to the presence of Ca-alginate hydrogel. The ESP can be identified from the small dark green dots in the image. This image suggests that the plant roots were able to grow through the alginate hydrogels with ESP without any harm or modification. This claim was further investigated using the SEM image of the plant root surface and root cross-section (Figure 2c–f). The SEM images of the root surface show that the Ca-alginate with ESP is attached to the root surface and around the root hair. The root hair length varied close to 45–65 µm. Figure 2d–f represent the cross-sectional SEM image of the plant root surrounded by the hydrogel with ESP. The images suggest the development of a vascular cylinder and xylem at the center of the plant root, although its internal structure is not well-defined due to the lyophilization process. However, the distinction between the root and Ca-alginate hydrogels with ESP is clearly visualized from the images. A zoomed image from Figure 2e shows the presence of ESP in the Ca-alginate hydrogels, as seen in Figure 2f. To differentiate the components present in the image, Figure 2f is pseudo-colored. The blueish-green dots were used to identify the presence of eggshell powder in the alginate hydrogels, and the olive-gray color was used to define the root in the image. From the SEM in Figure 2f, the alginate hydrogels have a microporous structure, which would have resulted from the lyophilization of the sample. Figure 2f also suggests that the eggshell powder is distributed randomly [46].

To visualize the effect of the eggshell powder and gel type on plant development, lettuce growth was observed in terms of leaf area incrementation and increase in shoot length. A very low concentration (0.1 wt.%) of eggshell powder was used and mixed with the ‘Coarse’ and ‘Fine’ hydrogels. The growth was monitored daily until day 8. 

**Figure 3:**
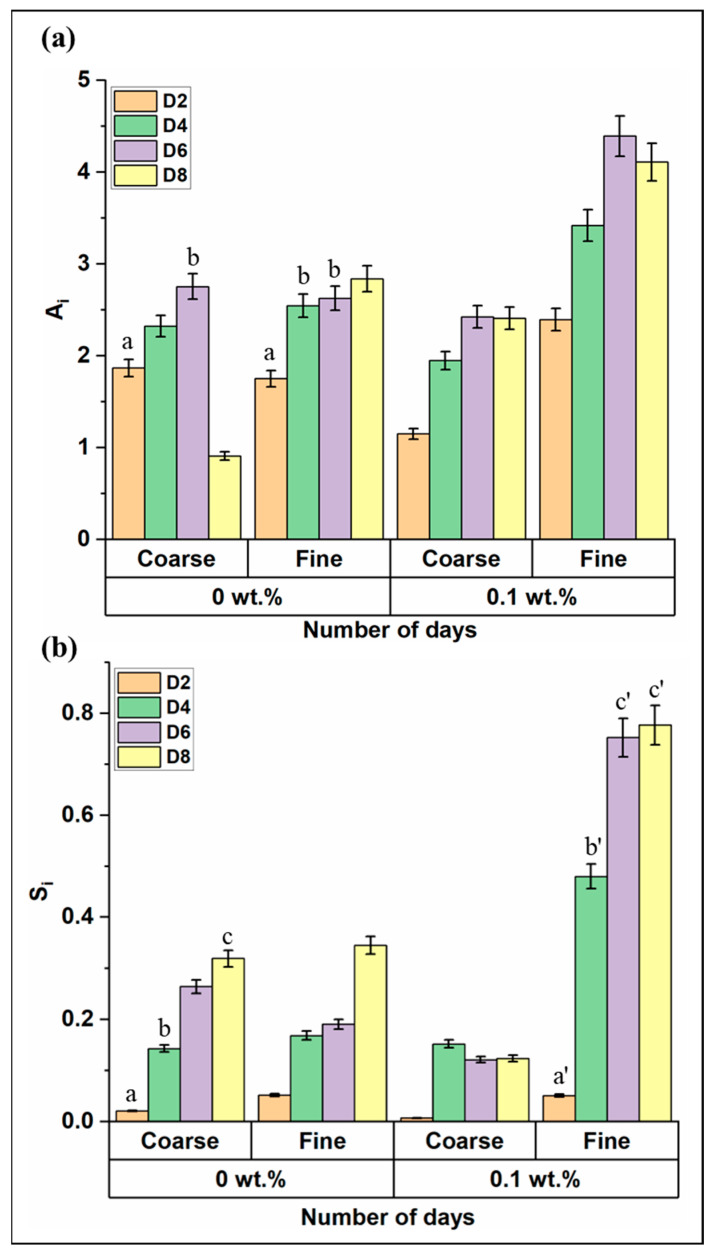
Plant growth with a lower concentration of ESP mixed with Ca-alginate hydrogel. (**a**) Plant leaf area incrementation (A_i_) with bare hydrogels and low concentration of eggshell powder till day 8; (**b**) Plant shoot length increase (S_i_) with bare hydrogels and low concentration of eggshell powder till day 8. Samples marked by different letters are significantly different from one another, while those marked by the same letter are not significantly different (*p* < 0.05; *n* = 3, Students *t*-test was performed).

For the hydrogels without the eggshell powder, the lettuce grown in fine hydrogels had a positive impact on the plant leaf area growth. For the coarse hydrogels, the plants grew into small well, well-defined green leaves by day 2 and continued to grow until day 6 (*p* < 0.05). After day 6, two out of three lettuce plants grown in coarse hydrogels wilted over time. This can be viewed from the decreasing trend in A_i_ (Figure 3a). The shoot length increase, S_i_, can be seen in both sets of plants grown in coarse and fine hydrogels. Even though plants showed increased shoot length in coarse hydrogels, A_i_ was declining. The reason could be due to osmotic stress in the plants, which led to wilting of the leaves in coarse hydrogels. In coarse and fine hydrogels with the ESP, the plants showed growth in the leaf area. The plants in coarse hydrogels showed an increase in leaf area and an almost constant trend in shoot length. Here, only one plant out of three started to wilt from day 4. This could be due to an error while planting the seedlings into the grow basket. Plants in fine hydrogels showed an upward trend in both A_i_ and S_i_ (*p* < 0.05). The reason for this increased trend could be that eggshell powder can be a source of nutrients and support. This could further be related to the studies performed by Vu et al., where the addition of eggshell powder showed improved growth characteristics in groundnut cultivation [34]. Fine hydrogels having smaller gel lengths could have led to the increased scope for water and nutrient availability to the plants. 

**Figure 4:**
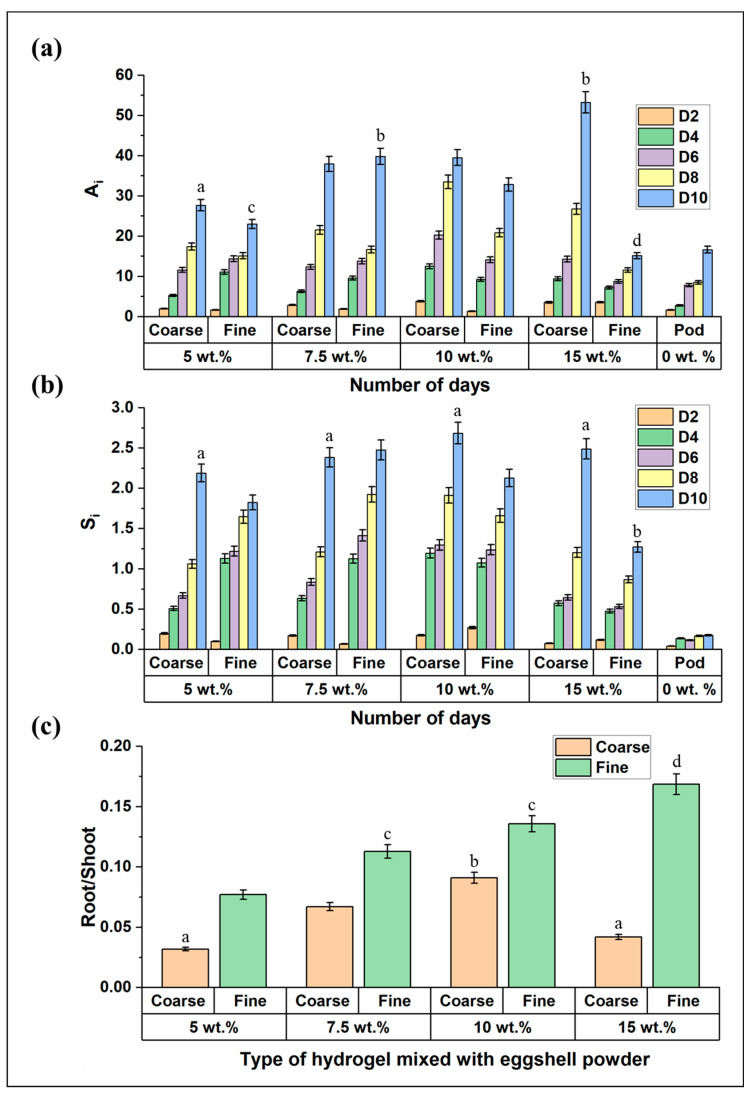
Plant growth with a higher concentration of eggshell powder (ESP) mixed with Ca-alginate hydrogel. (**a**) Plant leaf area incrementation (A_i_) until day 10. (**b**) Plant shoot length increase (S_i_) until day 10. (**c**) Root-to-shoot ratio of fresh plant at day 15. Samples marked by different letters are significantly different from one another, while those marked by the same letter are not significantly different (*p* < 0.05; *n* = 3, one-way ANOVA and students *t*-test were performed).

A very low concentration of eggshell powder mixture in the fine hydrogels showed better growth in the lettuce plants. Hence, the concentration of eggshell powder was increased with fine and coarse hydrogels to monitor the plant growth based on leaf area incrementation, shoot length increase, and root-to-shoot ratio of the plants. The eggshell powder concentration was arbitrarily chosen as 5, 7.5, 10, and 15 wt.% to be mixed with the two types of hydrogels. Here, the leaf area incrementation (A_i_) and shoot length increase (S_i_) were monitored for 10 days. The addition of higher eggshell powder concentration yielded higher leaf and shoot lengths in lettuce plants compared to the lower eggshell powder concentration in plants [34]. For 5 and 7.5 wt.% ESP concentration in gels, the A_i_ in the coarse and fine gels were found to be similar (Figure 4a). Furthermore, lettuce grown in the 15 wt.% ESP concentration in coarse hydrogels had better A_i_ than the other available growth conditions. The reason could be the nutrients obtained from the higher concentration of ESP mixed with the gel. Previous studies also showed that the addition of a higher concentration of ESP facilitated plant growth and provided nutrients [47,48]. On the other hand, the fine hydrogels with the same ESP concentration showed lower A_i_. As the plant grows, it requires increasing water and nutrients; roots are the source of the water and nutrient transfer to the plant. For fine 15 wt.% ESP, the smaller length of the gels and the high concentration of ESP could have caused the lettuce roots to be densely packed. This further contributed to the plants’ absence of water and nutrients, limiting aeration in the roots, thus leading to the lowest growth of leaf area, among other conditions. The plants grown in growing pods also showed lower A_i_ due to the lower water spray time than the conventional water spray time in aeroponic systems. Out of three specimens, one of the plants leaves wilted around day 4. The lower water spray time could have caused water deficiency in the plants, which caused the wilting and lower growth rate of plants. 

One way ANOVA test was performed on day 10 for the leaf area incrementation, A_i_; it was found that the effect of concentration (*p* = 9.91 × 10^−8^) and gel type (*p* = 4.57 × 10^−2^) were highly significant for the plant area increase. For the differences in leaf area incrementation, the student’s ‘*t*’ test comparing two groups showed that the A_i_ was insignificant for 5, 7.5, and 10 wt.% ESP concentration (Table S1) for coarse hydrogels and significant for 15 wt.% ESP concentration with coarse hydrogels compared to other ESP concentrations (*p* < 0.05). The A_i_ for the leaves grown in 15 wt.% ESP mixed with coarse and fine hydrogels were significantly different (*p* < 0.025). However, the A_i_ for the leaves grew by 7.5% and 10 wt.% ESP with fine hydrogels and 15 wt.% ESP with coarse hydrogels were insignificant (Table S1). 

Afterward, the shoot length increase in the plants was almost the same for both coarse and fine hydrogels with ESP concentration of up to 10 wt.% (Figure 4b). However, S_i_ was much lower for the plants grown in 15 wt.% ESP fine hydrogels and in grow pods compared to the other conditions. The S_i_ followed the same trend as the A_i_. The lower S_i_ would be related to the water and nutrient deficiency of the plant roots. This was also seen in the student’s *t*-test on day 10, which showed significant differences between the shoot length fine hydrogel with 15 wt.% ESP concentration and coarse hydrogels with all ESP concentrations and (*p*-values in Table S2).

Additionally, the root-to-shoot ratio (RSR) of the fresh plants can be viewed from Figure 4c. RSR was measured on day 15 after planting the seedlings. The higher RSR of the fresh plant would suggest increased root weight compared to the shoot weight. The plants grown in fine hydrogels exhibited a higher RSR than those grown in coarse hydrogels of the same ESP concentration. It can also be seen that out of the plants grown in fine hydrogels with different higher concentrations of eggshell powder, the plants grown in fine hydrogels with 15 wt.% showed the highest RSR. This indicated lower shoot growth, which can also be seen from the lower A_i_ and S_i_ values in Figure 4a,b. Among the plants on coarse hydrogels with different higher concentrations, the 5 wt.% and 15 wt.% ESP showed smaller and similar RSR ratios. 

The ANOVA test for the RSR data showed significant differences in size (*p* = 6 × 10^−6^) and concentration (*p* = 0.016) of plants grown in the gels, and the student *t*-test showed that plants in the 5 and 15 wt.% ESP-mixed hydrogels showed similar and smaller RSR ratios compared to other samples. Even though the area increase (A_i_) was insignificant for 15 wt.% ESP with coarse hydrogels and 7.5 and 10 wt.% ESP with fine hydrogels, the RSR values were significantly different (Table S3). The statistical data and graph indicated that 15 wt.% ESP-mixed coarse hydrogels were ideal for lettuce growth in the aeroponic system. Therefore, they were selected for further studies.

**Figure 5:**
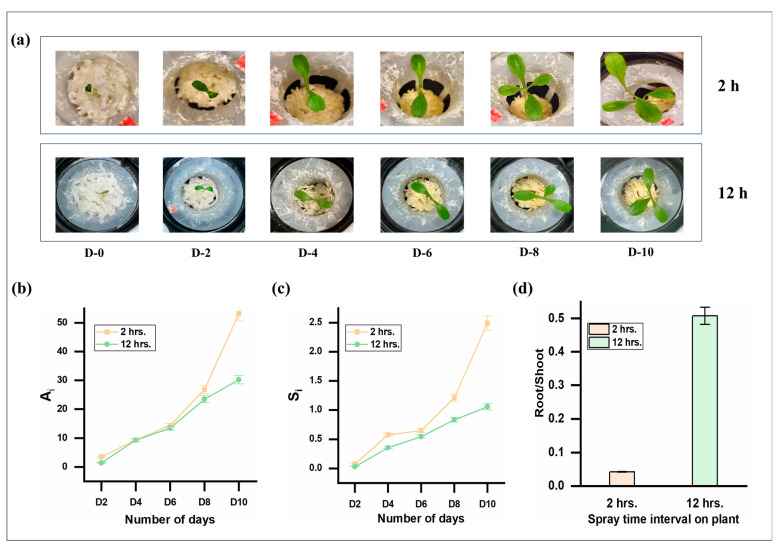
Effect of the spray time interval for lettuce growth. (**a**) Growth of romaine lettuce over time in response to spraying time interval until day 10. (**b**) Plant leaf area incrementation (A_i_) in response to spraying time interval until day 10. (**c**) Plant shoot length increase (S_i_) in response to spraying time interval until day 10. (**d**) Root-to-shoot ratio of fresh plant at day 15 in response to spraying time interval.

In the previous experiment shown in Figure 4a–c, the plants treated with coarse hydrogel containing 15 wt.% ESP and sprayed with a 2 h interval showed better growth in terms of leaf area incrementation, shoot length increase, and root-to-shoot ratio. To identify the potential impact of extending the spray time interval, another set of experiments was conducted with a 12 h interval. All other growth conditions remained constant. The growth pattern can be seen in Figure 5a, where on day 2, the leaves of the 2 h spray time interval plants looked more prominent, and on days 4 and 6, the leaves looked similar for plants in both conditions. However, from day 8 and onwards, it was noted that the leaves grown with a 2 h spray time interval exhibited a relatively broader and more dispersed morphology when compared to those subjected to a 12 h spray time interval. This can be further seen from the area incrementation (A_i_) for both 2 h and 12 h spray time intervals, which were similar until day 6, as shown in Figure 5b. After day 6, A_i_ was lower in the plants grown with a 12 h spray time interval. As plants grow, their water requirements increase [49]. In the first six to seven days of both sets of experiments, the available moisture in the hydrogels could have mitigated the water requirement for the initial growth of plants, resulting in a similar growth trend for both A_i_ and S_i_ irrespective of spray time interval, as shown in Figure 5b,c. However, after day 6, the plants required more water, leading to lower A_i_ and S_i_ for the 12 h spray time interval. As seen in Figure 1a, the gel’s WRC capacity drops to ~89–92%, causing a water content drop to almost 11% for the first 12 h. The regular 12 h spray time interval could have caused further drying in the gels, causing water stress in the plant roots, thus contributing to lower plant shoot growth and higher root growth [50,51]. Likewise, the root/shoot ratio (RSR) of the fresh plants also showed a higher value close to 0.5 for the 12 h spray time interval, indicating water deficiency in the roots and lesser shoot growth, as depicted in Figure 5d. Thus, the spray time interval plays an essential role in the growth of plants in Ca-alginate hydrogel.

**Figure 6:**
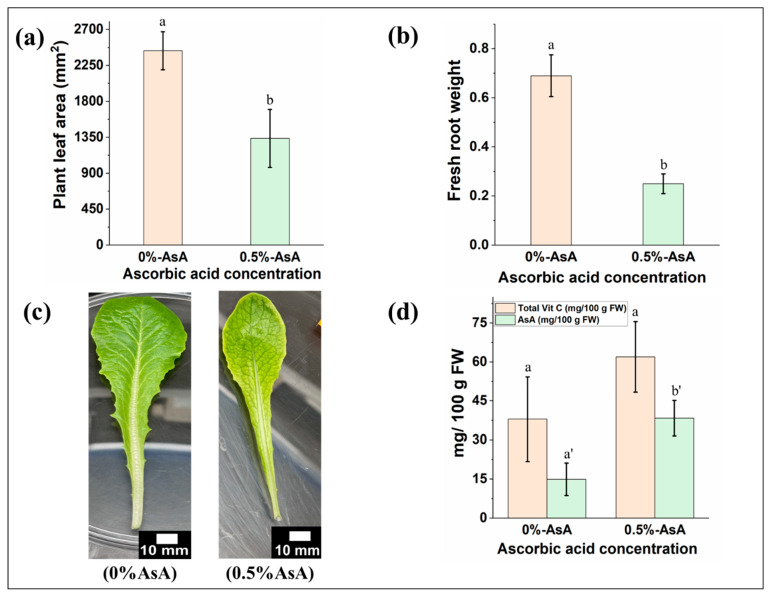
Plant growth with and without ascorbic acid treatment. (**a**) Plant leaf area after different concentrations of ascorbic acid treatment. (**b**) Plant root weight after different concentrations of ascorbic acid treatment. (**c**) Plant leaf after different concentrations of ascorbic acid treatment. (**d**) Total Vitamin C and AsA concentration after different concentrations of ascorbic acid treatment. Samples marked by different letters are significantly different from one another, while those marked by the same letter are not significantly different (*p* < 0.05; *n* = 3, Students *t*-test was performed).

As the gels showed better plant growth in the given conditions, and the Ca-alginate hydrogels have been previously used for the nutrient transfer to the plants, they were subjected to be analyzed for the biofortification of vitamin C in lettuce. The lettuce grown without ascorbic acid was greener and denser than leaves with a 0.5% concentration (Figure 6c and Figure S2). The growth rate of the leaves was lower in the 0.5% ascorbic acid (AsA)-treated sample compared to the leaves grown without any treatment (Figure S2a,b). Three fresh leaves from each set of samples were chosen for area analysis. The effect of ascorbic acid treatment on the leaf area can be seen in Figure 6a. The leaf area is highest in the absence of ascorbic acid in the gels compared to 0.5% AsA-treated plant leaves (*p* < 0.025). A similar trend could also be seen for the root weight sample as well in Figure 6b (*p* < 0.01). The root weight was higher in plants without AsA, and the AsA-treated plants showed lower root weight. One plausible explanation for these findings is the presence of a significant amount of AsA in the plants, contributing to an increase in electrical conductivity, thus inversely affecting plant growth [52,53,54]. Also, another probable explanation could be the difference in osmotic stress in plant roots caused by the surrounding AsA-absorbed ESP mixed Ca-alginate gels, which eventually caused reduced nutrient–water uptake from the gels [52]. Nevertheless, the plants did not wilt over time, and the leaves looked fresh after five weeks (Figure S2). The total vitamin C and ascorbic acid concentrations were measured for the leaves grown without any treatment and 0.5% AsA. Three samples from each set were chosen for the analysis. The total vitamin C concentration in leaves without AsA is 38.02 ± 2 mg/100 g FW, and with 0.5% AsA, it is 61.94 ± 3 mg/100 g FW (*p* < 0.1). The AsA concentration in the lettuce with 0.5%-AsA showed a higher AsA concentration compared to the plants without any AsA (Figure 6d, *p* < 0.025). This indicates that the application of AsA made a positive impact on the increase of AsA in the plants.

The plants with the AsA treatment showed lower values compared to plants grown in soil with ascorbic acid treatment, where the AsA concentration was 67.66 mg/100 g FW, whereas in our samples, the value was 38.35 mg/100 g FW [55]. The total vitamin C concentrations were not significantly different (*p* < 0.1) for plants grown in Ca-alginate hydrogels with 15 wt.% ESP without AsA and 0.5% AsA absorbed Ca-alginate hydrogels with 15 wt.% ESP. One of the probable reasons could be due to the small number of replications used in the test, which might have caused the deviation to be higher. Another reason for this similar concentration with the non-treated samples could be the release of ascorbic acid from the gels during the nutrient water spray intervals. The nutrient water sprays could have diluted the existing AsA concentration in the gels over time. The conventional AsA treatment would require AsA application to the plants at some intervals, thus obtaining higher vitamin C concentration. To increase the vitamin C concentration in plants, periodic ascorbic acid (AsA) treatment is necessary. In the experiment, AsA was only initially absorbed by the gels, and no external AsA was introduced thereafter. However, the plants treated with 0.5% AsA absorbed Ca-alginate hydrogels with 15 wt.% ESP showed four to five times better vitamin C concentration than the conventional soil-based biofortification [56]. Although the vitamin C concentration is similar in the two conditions after the experiment, it allows for exploring the scope of incorporating vitamin C into the plant leaves. Certain modifications could be made to tune the concentration to achieve the desired goal. 

## 3. Conclusions

In this study, eggshell powder (ESP) mixed with calcium–alginate hydrogel was used to grow romaine lettuce in an aeroponic system. The type of hydrogels and the eggshell powder concentration showed a positive impact on the growth of the plants. The studies showed that the Ca-alginate use in the aeroponic system has increased water efficiency by minimizing the spray time compared to the conventional aeroponic system. The spray time interval for the lettuce growth also showed differences in the leaf area, shoot length, and root/shoot ratio. These experiments were followed by promoting the biofortification of vitamin C in the lettuce leaves. The results showed a better ascorbic acid concentration in the plant leaves. The vitamin C concentration was similar in plants treated with ascorbic acid and without ascorbic acid. In the future, further research should be conducted to improve the ascorbic acid treatment method while growing the plants. The biofortification of vitamin C in these leaves creates a scope for the biofortification of other available nutrients necessary for human health development. 

## 4. Materials and Methods

### 4.1. Materials

Sodium alginate (viscosity: 4–12 cP, 1% in H_2_O (25 °C)) was purchased from Sigma-Aldrich, calcium chloride (Desiccant, 20 mesh and finer, MW 110.98) from Fisher Chemical, and eggshell powder (Calcium (90%), Magnesium (6%), Phosphorus (1%)) from Natural Innovative Solutions, Albany, OR, USA. Romaine lettuce seeds were purchased from Amazon (Gardner Basics), miracle-gro water-soluble all-purpose plant food was purchased from Amazon (NPK value is 24-8-16). For the Vitamin C analysis, ascorbic acid (L-(+)-Ascorbic acid, Alfa Aesar, Haverhill, MA, USA) was used with KOH (Thermo Fisher Scientific, Waltham, MA, USA) as a buffer. 

### 4.2. Experimental Setup

The experiments were conducted in a controlled environment in the laboratory. The experiments were conducted at room temperature (18–24 °C), and the relative humidity of the plant growth chamber was maintained by an RH controller (Inkbird digital Wi-Fi humidity controller IHC-200) at 75% ± 5%. The experiments were conducted with supplemental LED (FECiDA 600W LED Grow Light) light at a PPFD value of approximately 650–700 µmol m^−2^ s^−1^ with 10 h of photoperiod. 

For each set of experiments, the seeds were selected at a weight range of 0.9–1.4 mg and germinated in a closed container on a damp paper towel for 4 days. The romaine lettuce seeds were planted in hydrogel and placed on grow baskets on the 5th day of germination. In each pod, 1–5 seedlings were planted, and the distance between each pod was 2.3 cm. These pods were then put in a plastic grow chamber (0.246 m (L) × 0.362 m (W) × 0.229 m (H)) (Viagrow VCLN24 Clone Machine), which had a water container inside it with a clone pump (Viagrow). Water was sprayed on the hydrogels for 10 s every 2 h. The plant nutrients were added to the DI water with a target of 150 ppm N concentration. 

### 4.3. Plant Measurement

The plant data were taken in triplicate for each condition. After the seedling were planted into the hydrogel, the leaf images were collected every day until day 8/10. The seedling plantation day was termed as day 0. Leaf area was also measured from these images. The shoot length of the plants was measured from day 1 to day 8/10. Here, the leaf area incrementation from the initial day of planting the seedling to a specific day, ‘D’, was used to measure plant growth. This is termed A_i_. Another method of measuring plant growth involved recording the increase in shoot length from day one until a specific day, ‘D’. This is termed S_i_. The equations are mentioned as follows:

(1) $$A_{i}=\frac{\text{Leaf area at day ‘D’}-\text{Leaf area at day ‘0’}}{\text{Leaf area at day ‘0’}}$$

(2) $$S_{i}=\frac{\text{Leaf shoot length at day ‘D’}-\text{Leaf shoot length at day ‘1’}}{\text{Leaf shoot length at day ‘1’}}$$

On day 15, the plants were taken out of the hydrogels, and the roots were cleaned with DI water and separated from the shoot by a disinfected scalpel. The fresh weight of the root and shoot were measured for all plants to determine the fresh root/shoot ratio of the plants. 

### 4.4. Preparation of Eggshell Powder Mixed with Alginate Hydrogel

For the preparation of alginate hydrogel, 2 wt.% alginate solution was prepared, and for crosslinking, 1, 2, and 3 wt.% CaCl_2_ solution was prepared. A 30 cc BD- syringe was used to draw the alginate solution and then pour it into the CaCl_2_ solution. The crosslinking time was set to 10 min, and the gel dispersed in the gel was stirred intermittently at this time. After the crosslinking, the gel was separated from the CaCl_2_ solution with a strainer and then washed with DI water up to 3 times. 

After the gel preparation, the selected hydrogel was divided into two categories—Coarse and Fine—based on the length of the gel. The alginate hydrogels with a length ranging 20–40 mm were termed ‘Coarse’ gel, and the gel length ranging 3–10 mm was termed ‘Fine’ gel. A scalpel was used to cut the gels according to the desired length (Figure S1b,c).

The eggshell powder was heated at 200 °C for 15–20 min to remove bacteria. Then, it was ground to a fine powder in a high-speed multifunctional grinder. This eggshell powder was mixed with the prepared alginate hydrogels at 0.1, 5, 7.5, 10, and 15 wt.% (Figure S1d,e).

### 4.5. Water Retention Capacity

Freshly prepared 1, 2, and 3 wt.% CaCl_2_ crosslinked hydrogels and the 1, 2, and 3 wt.% CaCl_2_ crosslinked hydrogels mixed with 7.5% ESP were put into a closed chamber at 75% ± 5% RH. The RH was chosen to replicate the plant growth RH condition. The initial weight of the samples was measured. The water retention capacity was measured after 2 h and 12 h.

The water retention capacity (WRC) value was calculated using the Equation (3). The initial weight of the hydrogel is termed W_i_, and the hydrogel weight at the stated time interval is termed W_t_.

(3) $$WRC=100-(100\times \frac{W_{i}-W_{t}}{W_{i}})$$

### 4.6. Water Uptake Capacity

The 1, 2, and 3 wt.% CaCl_2_ crosslinked hydrogel weights were measured after 2 h and 12 h after keeping them in the closed chamber at RH 75% ± 5%. Then, DI water was sprayed on the hydrogels inside the chamber for 10 s to replicate the water spray time of the romaine lettuce plants. After spraying the gels with DI water, excessive water was removed from the gel, and the weights were measured. The initial weight of the hydrogel after a certain time was termed W_t1_, and after water spraying, the hydrogel weight was termed W_s_. The water uptake (WU) test of the hydrogels was calculated from Equation (4)

(4) $$WU=100\times \frac{W_{s}-W_{t1}}{W_{t1}}$$

### 4.7. Scanning Electron Microscopy (SEM) Analysis

The scanning electron microscopy (SEM) analysis was performed using a scanning electron microscope (Hitachi 3400N VP-SEM and Zeiss Crossbeam 540 FIB-SEM). The plant roots with hydrogel were put in small tubes with DI water. Then, the samples were flash frozen by liquid nitrogen and immediately put in a freeze-dryer at −80 °C. After freeze-drying, the samples were coated with an iridium sputter coating before the analysis. The image analysis was performed at 3 kV and 5 kV.

### 4.8. Fluorescence Microscopy

All fluorescence images were taken using Leica DMI 6000B microscope. Green fluorescence protein (GFP) images were taken after staining the samples with CellTracker™ Green CMFDA Dye (Thermo Fischer Scientific, Waltham, MA, USA) for 30 min, followed by 3 washes with phosphate buffer saline (PBS) to remove excess dye. GFP images were taken at a wavelength of 488 nm and exposure time of 200 ms.

### 4.9. Statistical and Image Analysis

The experiments were performed in triplets, and one-way ANOVA and student *t*-test (*n* = 3) were performed in Python and Excel, respectively. 

For the plant leaf area measurement, the image analysis was performed using ImageJ 1.53t software and Adobe Photoshop. For the area, shoot, and root/shoot ratio, the error bars were measured as a ±5% measurement error. 

### 4.10. Vitamin C Extraction and Analysis

For the Vitamin C test, 0.5% ascorbic acid solutions were prepared. To neutralize the solution up to pH 6, KOH was mixed into the solution [54]. 

To prepare the ascorbic acid-absorbed Ca-alginate hydrogels, 2 wt.% Ca-alginate hydrogels were freshly prepared. The excessive water was removed from the gel using a strainer, and the gels were soaked in the ascorbic acid-KOH solution for two days. After this, the germinated romaine lettuce seedlings were planted on Ca-alginate hydrogel with 15 wt.% ESP, and ascorbic acid-absorbed Ca-alginate hydrogel with 15 wt.% ESP. Five seedlings were planted in one grow basket containing hydrogels. The growth was monitored, and the leaves were harvested in week 5 for the Vitamin C analysis. 

The Vitamin C extraction of the lyophilized leaves was performed using 6% trichloroacetic acid adapted from Sérino et al. [57]. The analysis was performed using microplate spectroscopy [57]. After the extraction, 20 μL of extracts were distributed in a microplate, and the oxidized ascorbate was reduced using a reducing agent dithiothreitol (DTT) for the determination of vitamin C. Simultaneously, ascorbic acid (AsA) determination, 0.5 M phosphate buffer, pH 7.5 was used instead of DTT. The absorbance of the prepared samples was read at 550 nm using a microplate spectrophotometer (SpectroMax iD3, San Jose, CA, USA). Total vitamin C and AsA were determined in mg/ 100g FW (fresh weight) using the equation mentioned in Sérino et al. [57].

## Data Availability

All data and materials are available on request from the corresponding author.

## Supplementary Materials

The following supporting information can be downloaded at: https://www.mdpi.com/article/10.3390/gels10050322/s1, Figure S1: (a) Preparation of alginate hydrogel, (b) Freshly prepared coarse hydrogel, (c) Freshly prepared fine hydrogel, (d) Freshly prepared coarse hydrogel with 15 wt.% eggshell powder, (e) Freshly prepared fine hydrogel mixed with 15 wt. % eggshell powder; Figure S2: (a) Leaves without ascorbic acid, (b) Leaves with 0.5% ascorbic acid; Table S1: Student’s t-test results for hydrogels with different ESP concentrations for leaf area increase; Table S2: Student’s t-test results for hydrogels with different ESP concentrations for shoot length; Table S3: Student’s t-test results for hydrogels with different ESP concentrations for RSR
