# Supplementary material for: Growth of Romaine Lettuce in Eggshell Powder Mixed Alginate Hydrogel in an Aeroponic System for Water Conservation and Vitamin C Biofortification
Source: Gels. 2024 May 9;10(5):322. doi: 10.3390/gels10050322 (PMC11121658; doi:10.3390/gels10050322)
Supplement: Supplementary file 1 [file gels-10-00322-s001.zip › gels-2990498-supplementary.pdf]

Supporting Information

Growth of Romaine Lettuce in Eggshell Powder Mixed Alginate Hydrogel in an Aeroponic System for Water Conservation and Vitamin C Biofortification

Fariha Afnan,[1] Md Nayeem Hasan Kashem,[1] Rutwik Joshi,[1] Catherine Simpson,[2]* Wei Li [1]*

1. Department of Chemical Engineering, Texas Tech University, Lubbock, TX, United States
2. Department of Plant and Soil Science, Texas Tech University, Lubbock, TX, United States

* Corresponding authors: Catherine.simpson@ttu.edu and wei.li@ttu.edu

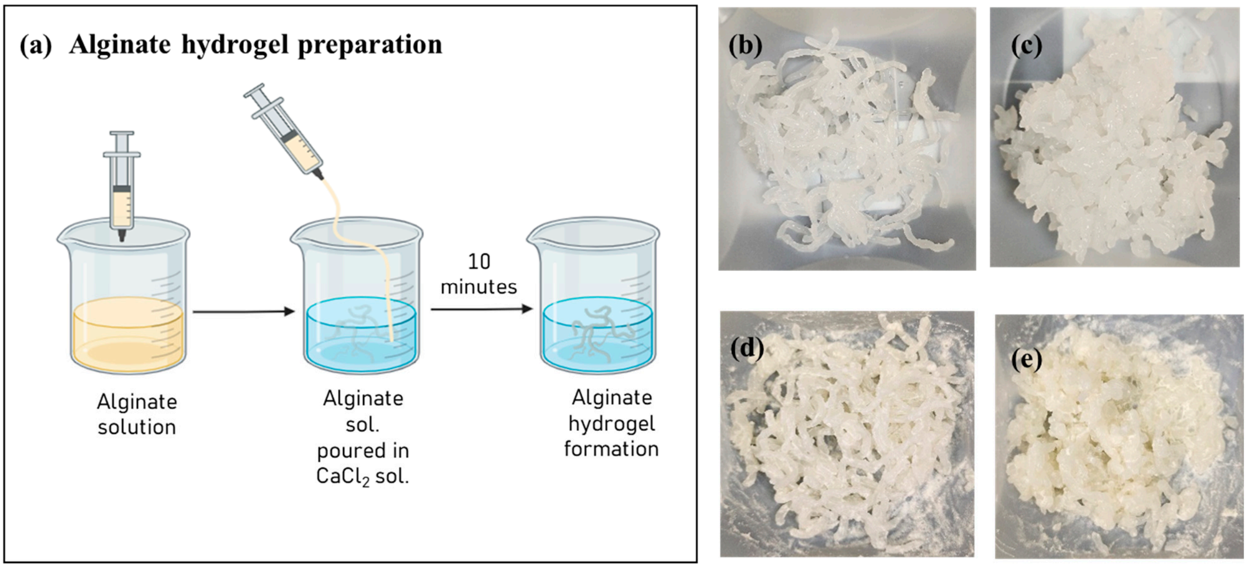

Figure S1: (a) Preparation of alginate hydrogel, (b) Freshly prepared coarse hydrogel, (c) Freshly prepared fine hydrogel, (d) Freshly prepared coarse hydrogel with 15 wt.% eggshell powder, (e) Freshly prepared fine hydrogel mixed with 15 wt. % eggshell powder.

(a) 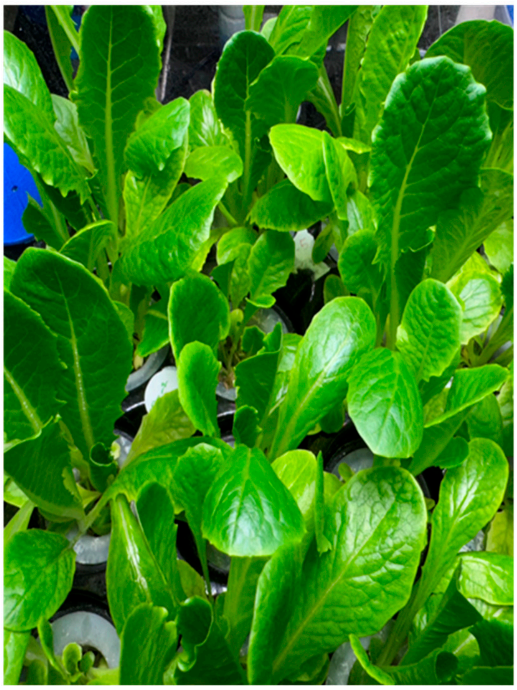 (b) 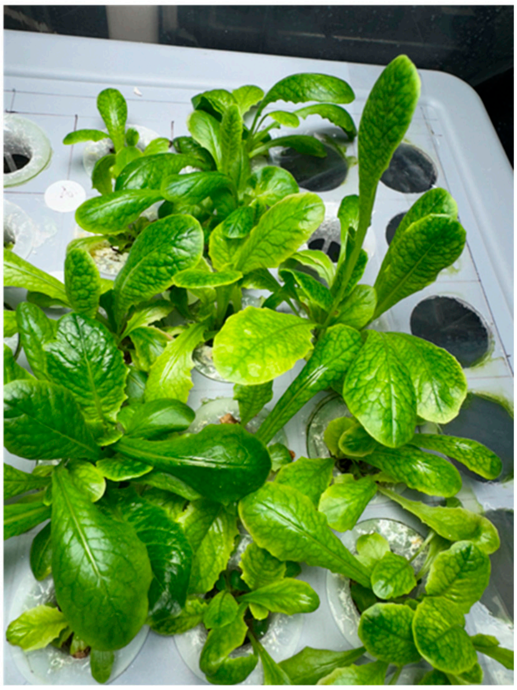

Figure S2: (a) Leaves without ascorbic acid, (b) Leaves with 0.5% ascorbic acid.

Table S1: Student's t-test results for hydrogels with different ESP concentrations for leaf area increase

Parameters ESP Conc. (wt.%)	t	$t_{critical}$	P	Null Hypothesis
**5, 7.5 (C/C)**	1.538	1.19	0.15	not void
**5, 10 (C/C)**	0.644	0	0.5	not void
**5, 15 (C/C)**	2.767	2.132	0.05	void
**15, 5 (C/F)**	3.107	2.776	0.025	void
**15, 7.5 (C/F)**	0.6302	0	0.5	not void
**15, 10 (C/F)**	1.25	1.19	0.15	not void
**15, 15 (C/F)**	5.628	4.604	0.005	void

Note: C/C = Comparison between Coarse and Coarse hydrogel

C/F= Comparison between Coarse and Fine hydrogel

Table S2: Student's t-test results for hydrogels with different ESP concentrations for shoot length

Parameters	t	$t_{critical}$	P	Null Hypothesis
**ESP Conc. (wt.%)**				
**15, 5 (F/C)**	2.969	2.776	0.025	void
**15, 7.5 (F/C)**	2.629	2.132	0.05	void
**15, 10 (F/C)**	3.766	3.747	0.01	void
**15, 15 (F/C)**	5.757	4.604	0.005	void

F/C= Comparison between Fine and Coarse hydrogel

Table S3: Student's t-test results for hydrogels with different ESP concentrations for RSR

Parameters	t	$t_{critical}$	P	Null Hypothesis
**ESP Conc. (wt.%)**				
**5, 7.5 (C/C)**	3.647	2.776	0.01	void
**5, 10 (C/C)**	7.063	4.604	0.005	void
**5, 15 (C/C)**	0.803	0.741	0.25	not void
**15, 5 (C/F)**	2.065	1.533	0.1	not void
**15, 7.5 (C/F)**	5.096	4.604	0.005	void
**15, 10 (C/F)**	4.58	4.604	0.005	void
**15, 15 (C/F)**	5.969	4.604	0.005	void
